# Accounting for center in the Early External Cephalic Version trials: an empirical comparison of statistical methods to adjust for center in a multicenter trial with binary outcomes

**DOI:** 10.1186/1745-6215-15-377

**Published:** 2014-09-26

**Authors:** Angela Reitsma, Rong Chu, Julia Thorpe, Sarah McDonald, Lehana Thabane, Eileen Hutton

**Affiliations:** Midwifery Education Program, McMaster University, 1280 Main St. W., MDCL 2210, Hamilton, ON L8S 4 K1 Canada; Agensys Inc, 1800 Stewart St, Santa Monica, CA 90404 USA; Department of Obstetrics & Gynecology, McMaster University, 1280 Main St. W. Room 3N52B, Hamilton, ON L8S 4 K1 Canada; Department of Clinical Epidemiology and Biostatistics, McMaster University, Biostatistics Unit, St. Joseph’s Healthcare Hamilton, 3rd Floor, Martha Wing, Room H-325, 50 Charlton Ave, E, Hamilton, ON L8N 4A6 Canada

**Keywords:** Randomized controlled trials, Center effect, Multicenter trials, Trial design, Trial analysis, Random effect, Mixed-effects, Generalized estimating equations, Generalized linear mixed model

## Abstract

**Background:**

Clustering of outcomes at centers involved in multicenter trials is a type of center effect. The Consolidated Standards of Reporting Trials Statement recommends that multicenter randomized controlled trials (RCTs) should account for center effects in their analysis, however most do not. The Early External Cephalic Version (EECV) trials published in 2003 and 2011 stratified by center at randomization, but did not account for center in the analyses, and due to the nature of the intervention and number of centers, may have been prone to center effects. Using data from the EECV trials, we undertook an empirical study to compare various statistical approaches to account for center effect while estimating the impact of external cephalic version timing (early or delayed) on the outcomes of cesarean section, preterm birth, and non-cephalic presentation at the time of birth.

**Methods:**

The data from the EECV pilot trial and the EECV2 trial were merged into one dataset. Fisher’s exact method was used to test the overall effect of external cephalic version timing unadjusted for center effects. Seven statistical models that accounted for center effects were applied to the data. The models included: i) the Mantel-Haenszel test, ii) logistic regression with fixed center effect and fixed treatment effect, iii) center-size weighted and iv) un-weighted logistic regression with fixed center effect and fixed treatment-by-center interaction, iv) logistic regression with random center effect and fixed treatment effect, v) logistic regression with random center effect and random treatment-by-center interaction, and vi) generalized estimating equations.

**Results:**

For each of the three outcomes of interest approaches to account for center effect did not alter the overall findings of the trial. The results were similar for the majority of the methods used to adjust for center, illustrating the robustness of the findings.

**Conclusions:**

Despite literature that suggests center effect can change the estimate of effect in multicenter trials, this empirical study does not show a difference in the outcomes of the EECV trials when accounting for center effect.

**Trial registration:**

The EECV2 trial was registered on 30 July 30 2005 with Current Controlled Trials: ISRCTN%2056498577.

## Background

The rise of evidence-based medicine has increased the number of randomized controlled trials (RCTs) conducted to test medical and surgical interventions [1]. Multicenter trials are often used to accumulate large sample sizes in a short time period, or to meet sample size requirements that would be impossible within one center. The inclusion of different centers and providers is beneficial in pragmatic trials as it allows for the greater generalizability of trial results. However, the potential variation between centers poses methodological issues. Each center needs to rigorously follow the study protocol, particularly around inclusion and exclusion criteria, and the application of the intervention to reduce heterogeneity and allow for outcome results to be pooled. Furthermore, accurate reporting of the characteristics of the centers involved in the study can allow readers to assess the risk of bias and the usefulness of the results [1, 2].

The assumption made in many multicenter trials is that participants recruited to the trial are independent of each other. This assumption of independence is necessary to apply routine statistical methods such as the Student’s t-test or chi-squared test. However, management of individuals within the same trial center may be similar, leading to the potential of outcomes from these individuals being correlated with each other. It is not hard to imagine that intervention success rates could differ from one center to the next due to any number of combinations of practitioner experience, nursing support and expertise, medical equipment, and center-specific treatment practices. When trial centers are in different international locales, the dissimilarities could be magnified. The correlation of outcomes among individuals within a study center is a type of clustering described as a center effect. If center effects are overlooked, incorrect effect estimates, confidence intervals, and *P* values may be the result [2, 3].

Many RCTs test non-pharmacologic treatments such as surgery, technical procedures, devices, rehabilitation, psychotherapy, behavioral interventions, and complementary and alternative medicine [4]. A review of all RCTs published in 2000 revealed that 10% of RCTs are for surgical or procedural interventions [5]. These trials have specific issues compared to pharmacologic trials because treatments are less standard and blinding is more difficult [4]. The group responsible for the Consolidated Standards of Reporting Trials (CONSORT) Statement published an extension of the Statement to specifically guide researchers involved in RCTs of non-pharmacologic treatments [4, 6]. The Statement identifies center characteristics such as provider skill and center volume that could impact patient outcomes. Since clustering of outcomes at study centers may reduce statistical power, the CONSORT group recommends accounting for clustering in sample size calculations and in statistical analyses [4].

Despite the development of statistical methods to account for center effect and the recommendations by trial reporting guidelines, evidence from reviews of the literature indicate that most individually randomized multicenter trials do not account for center effect [1, 7]. Biau *et al*. conducted a systematic review of the account of center and provider effects in large surgical and interventional (non-pharmacological) RCTs [1]. A total of 68 multicenter interventional randomized trials of more than 200 patients between the years 2000 and 2005 met the inclusion criteria. They found that stratification by center at randomization was reported in 38% of trials and analysis adjusted for center was reported in 6% of trials [1]. Tangri *et al*. published a similar systematic review of the literature to assess the extent of adjustment for center in RCTs of medicinal products [7]. They included 101 multicenter RCTs published in 2007 in four prominent medical journals. Of the 101 trials, 36% used random allocation stratified by center, and 18% adjusted for center in the statistical analysis [7]. Both reviews concluded that improvements to trial reporting regarding center effects are needed. However, the literature provides little guidance and does not suggest one preferred statistical method to account for center effects. In fact, there is a lack of evidence on which models perform best in various situations [8].

The objective of this secondary analysis was to use a combined dataset of the Early External Cephalic Version (EECV) trials to demonstrate the use of available statistical methods to account for center effects in multicenter trials. The EECV trials had many centers and the main outcomes were binary. We reviewed the literature for statistical approaches - both population-averaged and random-effects models - which might fit with these study characteristics [3, 9–12]. We estimated the effect of external cephalic version (ECV) timing (early or delayed) on the outcomes of cesarean section, preterm birth, and non-cephalic presentation at the time of birth without accounting for center, and then assessed the consistency of the results under different methods of accounting for center.

### The Early External Cephalic Version trials

ECV is an obstetric procedure undertaken during pregnancy to attempt to manually turn a fetus through the maternal abdomen from the breech (buttocks down) presentation to a cephalic (head down) presentation. The pilot and full EECV trials were multicenter RCTs aimed at investigating the effectiveness of beginning ECV earlier in pregnancy (conducted between 34 weeks and 0 days and 35 weeks and 6 days gestation) compared to the usual timing of ECV at full term (37 weeks and 0 days or beyond) on pregnancy outcomes [13, 14]. At the time of the EECV trials, nearly all fetuses in the breech presentation at term or at the onset of labor were born by cesarean section. The primary outcome was the rate of cesarean section and the secondary outcome was the rate of preterm birth. The study measured overall success of the ECV procedure by including the rate of non-cephalic presentation at delivery as another outcome.

Eligible participants were women with a singleton breech fetus at a gestational age of 33 weeks and 6 days to 35 weeks and 6 days. Participants were randomly assigned to have the first ECV procedure early or delayed, with stratification by center and parity to ensure approximately equal numbers of early and delayed ECVs at each center, as well as balance the number of multiparous women (a known predictor of ECV success) in each group at each center.

Ethical approval was obtained for both the pilot trial (The University of Toronto Office of Research Services) and the EECV2 trial (The University of British Columbia Clinical Research Ethics Board, reference number: C04-0348 and the Research Ethics Board of Hamilton Health Sciences Research Ethics Board, reference number: 07-122). Furthermore, ethical approval was obtained from each of the sites where the recruitment took place. Informed consent was obtained from each woman who was enrolled in the trial.

The planned analysis called for Fisher’s exact test to assess the relationship between the exposure (timing of ECV procedure) and the primary and secondary outcomes (cesarean section and preterm birth). The effects of the intervention were reported using relative risks (RR) and 95% confidence intervals (CI). Subgroup analyses were completed using logistic regression to test for interactions between baseline characteristics and treatment group for the primary and secondary outcomes. None were found to be significant.

The EECV pilot trial recruited 233 women from 25 centers in 7 countries. There were non-significant decreases in the rates of cesarean section (RR 0.90; 95% CI 0.76 to 1.08; *P* = 0.32) and non-cephalic presentation at birth (RR 0.86; 95% CI 0.70 to 1.05; *P* = 0.09) for women in the early ECV group. There was a non-significant increase in the rate of preterm birth for women in the early ECV group (RR 1.42; 95% CI 0.56 to 3.59; *P* = 0.31) [13]. The clinically important findings of the EECV pilot trial supported the funding of a full-scale RCT.

The EECV2 trial recruited 1543 women from 68 centers in 21 countries. Women in the early ECV group were less likely to have a non-cephalic presentation at delivery (RR 0.84; 95% CI 0.75 to 0.95; *P* = 0.002), but the decrease in the cesarean section rate remained statistically non-significant (RR 0.93; 95% CI 0.85 to 1.02; *P* = 0.12), and the trend for an increase in preterm birth was strengthened, though still not statistically significant (RR 1.48; 95% CI 0.97 to 2.26; *P* = 0.07) [14].

## Methods

**Figure 1:**
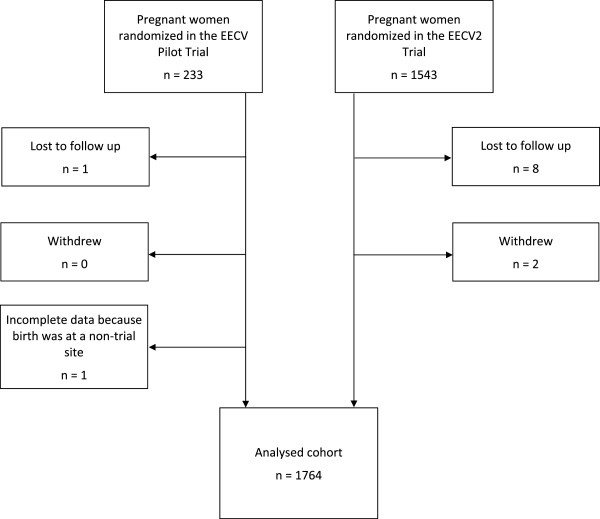
**Trial flow diagram**.

The data from the EECV pilot trial and the EECV2 trial were merged using SPSS IBM Corp., Armonk, NY, USA (version 19.0); SAS Institute Inc., Cary, NC, USA (version 9.2) and R Foundation for Statistical Computing, Vienna, Austria (version 2.12.2) were used for the statistical analyses. Participants were excluded from analysis if they withdrew from the trial, if they were lost to follow-up, or if there was missing data for the outcomes of interest: mode of delivery, gestational age at birth, or presentation at birth (cephalic versus non-cephalic) (Figure 1).

Using the merged dataset, we calculated the conditional maximum likelihood estimate of the odds ratio (OR) and the exact 95% CI based on non-central hypergeometric conditional likelihood using function ’exact2x2(…, tsmethod=”minlike”,…)’ in the R package ‘exact2x2’ [15, 16]. The CI matched the usual two-sided Fisher’s exact test which defines the *P*-value as the sum of probability of tables with smaller likelihood than the observed table.

We chose methods to account for center effect that functioned for data from a multicenter trial that used individual random allocation to group, stratified by center, with binary outcomes. The methods are broadly grouped as conditional and unconditional methods as follows. No further research ethics approval was required to conduct this secondary analysis.

### Conditional methods

Conditional methods estimate treatment effects by stratifying (or conditioning) on center. These methods are appropriate for multicenter trials in which participants are randomly assigned to different treatment arms within each center [3]. Conditional methods can be subdivided into fixed- and random-effects models. A fixed-effects analysis considers a center to represent only itself, while a random-effects analysis represents the population of centers from which the study sample was drawn [3, 17]. Accounting for center as both a fixed and random effect produces a single estimate of treatment effect if the treatment effect is assumed to be constant across centers. If the treatment effect is suspected to be different across centers, altering the model to include treatment-by-center interaction can improve the model’s fit [3]. Trials that test procedural interventions, such as the EECV trials, may be more likely to have heterogeneity of treatment effects across trial centers due to the difficulty of standardizing and administering the procedural intervention in a consistent manner across study sites [4]. Such heterogeneity may be reduced when a strict study protocol is followed [4]. The interaction that occurs when trial sites have different treatment effects is also known as effect modification.

We used six different conditional methods to account for center in the EECV trials. Four are fixed-effects approaches: i) the Mantel-Haenszel test, ii) logistic regression with fixed center effect and fixed treatment effect, iii) logistic regression with fixed center effect and fixed treatment-by-center interaction (weighted by center size), and iv) logistic regression with fixed intercept and fixed treatment-by-center interaction (un-weighted by center size). Two are random-effects models: v) logistic regression with random center effect and fixed treatment effect, and vi) logistic regression with random center effect and random treatment-by-center interaction. Models including treatment-by-center interaction as either fixed (iii and iv) or random effect (vi) allow for possible effect modification in the EECV trials. The models are described briefly below.

### Mantel-Haenszel test

The Mantel-Haenszel test is a fixed-effects analysis that summarizes data into a series of two × two tables based on covariates or strata. The Mantel-Haenszel test is often used when the trial has binary response variables and only two treatment groups; however, it has been generalized to analyze 2(response) × J(exposure) tables [18, 19]. The Mantel-Haenszel test performs well even with sparse data and is suitable for studies like the EECV trials that have many centers and few participants per center [3, 20]. The simplicity of the Mantel-Haenszel test is considered an advantage [20]. Centers that had only one participant were removed from the analysis. The statistical software program R Version 2.12.2 was used to run the Mantel-Haenszel chi-squared test.

### Logistic regression with fixed center effect and fixed treatment effect

Fixed-effects regression estimates within-center treatment effects [3]. It achieves this by including a separate intercept for each center as a fixed effect, restricting the inference of the results to included centers [8]. The model works best when there are many participants spread across few centers. Unreliable estimates can occur when the majority of centers have few patients and few events [3]. Since the EECV trials had low enrollment at some centers, the deletion of some small centers was expected for this model.

We used logistic regression with fixed center effect (Equation 2) to model the impact of treatment (*X*)(1 = early ECV*,* 0 = delayed ECV) on the odds of having the outcome event:
1

with a separate intercept for each center (*k*) as a fixed effect. Let *π*_*ik*_ = P (*Y* = 1 | *x* = *i*, *z* = *k*), for *i* = 1, 0; *k* = 1, …, 81. This is the probability of having the outcome event for someone receiving treatment *i*^*th*^ in the *k*^*th*^ center. *β*_0*k*_ represents the log odds for the control group in center *k*, and *β*_1_ represents the log odds ratio of the treatment across all centers:
2

### Logistic regression with fixed center effect and fixed treatment-by-center interaction (weighted and un-weighted by center size)

Most analyses assume the absence of effect modification. When effect modification is suspected or present, adding an interaction term allows estimation of treatment effect specific to each center. We used a fixed-effects logistic regression model with treatment-by-center interaction to account for the possibility that centers could have varying treatment effects (Equation 3). Here, *β*_1*k*_ represents the log odds ratio of having the outcome in the treatment group over the control group in the *k*^*th*^ center:
3

The model was run twice: once with and once without weighting by the size of the center (number of women enrolled). In multicenter trials with large disparities in center size, as was seen in the EECV trials, weighting by center size prevents small centers from inflating the variance [17]. The fixed-effects regression models were fitted using *proc genmod* in SAS version 9.2.

### Logistic regression with random-effect terms

Random-effects models are another way to model the hierarchical structure of patients within centers in individually randomized multicenter trials [21]. In contrast to fixed-effects models that provide results relevant only to the study sample, random-effects models are generalized to the entire population of possible centers by assuming that the trial centers are a random sample of all centers. Although this may not be the way that centers are chosen in a pragmatic RCT, the underlying notion is that the results of the trial provide probabilistic statements about patients in general, even those attending centers not included in the trial [17]. A random-effects model can be an improvement over a fixed-effects model when there are many centers [17, 22, 23].

Random-effects models have been used for decades for continuous outcomes, but model interpretation and fitting is more difficult with binary data. Random-effects models are also known by various other names such as: center-specific, mixed-effects, variance component, hierarchical, multistage, generalized linear mixed models, multilevel models, or empirical Bayes regressions depending on the context and subject area where the methods are applied [3]. In this manuscript, we refer to a statistical model including one or more random-effect terms as a general random-effects model. Two models incorporating random-effects were used to adjust for center in the EECV trials.

### Logistic regression with random center effect and fixed treatment effect

A logistic regression model with random center effect and fixed treatment effect, reflected in Equation 4, adjusts for center effects assuming that though there may be clustering of outcomes across treatment arms in each center, variation in the treatment effect is unlikely [21]. *β*_0_ is a fixed intercept representing the average log odds of experiencing an outcome in the control arm. *b*_0*k*_ is a random variable representing how the log odds in center *k* deviates from the overall log odds in the control arm. *b*_0*k*_ follows a Normal (0, σ^2^) distribution. The unknown parameter σ summarizes center heterogeneity in the outcome probabilities. *β*_1_ represents the single treatment effect over participating centers.
4

### Logistic regression with random center effect and random treatment-by-center interaction

This model includes an additional random effect *b*_1*k*_, often known as the random treatment-by-center interaction or random treatment effect at each center [8, 21]. By including random center effect and random treatment effect in the model (Equation 5), we account for center heterogeneity in log odds in the control group and variation in treatment effects across centers. (*b*_0*k*_, *b*_1*k*_) is assumed to follow a bivariate normal distribution specified in Equation 6, where  and  represent the variance of the random effects and *σ*_12_ = *σ*_21_ represents the covariance between *b*_0*k*_ and *b*_1*k*_.
56

We fit the random-effects logistic regression models using function *lmer() (lme4 package)* in R version 2.12.2. In a later version of R*, glmer()* has been introduced to replace *lmer()* for fitting generalized linear random-effects models. Refitting logistic regression models with random effects using *glmer()* in R version 2.15.2 produced the same results.

### Unconditional methods

Unconditional methods include marginal or population-averaged models that estimate an average treatment effect across all centers and then adjust for correlation of outcomes at centers [3]. One unconditional method, generalized estimating equations, was applied to the EECV trial data.

### Generalized estimating equations

Generalized estimating equations (GEE) model the marginal population treatment effects averaged across centers in two steps [11]. First a model similar to ordinary logistic regression without regard to the center is fitted. Then the model is refitted to adjust the standard error and CIs for within-center dependence. By using weighted combinations of observations, the GEE approach extracts the appropriate amount of information from correlated data [24]. Some studies suggest a large number of centers (for example, 30) is required for the underlying theory of the GEE model to apply [3, 12].

Application of the GEE model to the EECV trial data was achieved using data in the same individual patient-level format as was used for the random-effects regressions. The GEE model was run using *proc genmod* assuming exchangeable correlation structure in SAS Version 9.2. Intra-class (or intra-center) correlation (ICC) values were noted from the GEE output to present the magnitude of differences in treatment effect between the centers.

## Results

### Center characteristics

**Figure 2:**
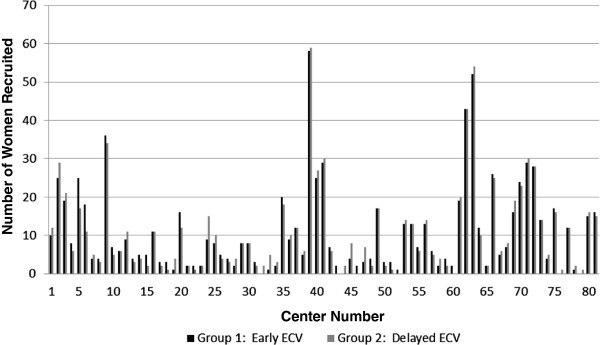
**Center recruitment and balance of stratification.** Center numbers: 1-22 (Canada); 23-28 (UK); 29-33 (USA); 34-46 (Australia); 47-51 (Israel); 52-53 (South Africa); 54-59 (Argentina); 60 (New Zealand); 61-65 (Chile); 66-67 (Denmark); 68 (Germany); 69 (Ireland); 70 (Jordan); 71-73 (The Netherlands); 74 (Poland); 75 (Spain); 76 (Brazil); 77 (Egypt); 78 (Portugal); 79 (Hungary); 80 (Estonia); 81 (Oman). ECV, external cephalic version.

The data from the EECV pilot trial and the EECV2 trial were merged to create one large dataset (Figure 1). A total of 81 centers from 22 countries contributed participants for the trials. Center sizes were unequal, with the number of women recruited at each center varying from 1 to 117. There were a small number of centers that recruited large numbers of participants, and a large number of centers that recruited small numbers of participants. The mean center recruitment was 45 and the median center recruitment was 13. Random block sizes ensured that approximately equal numbers of patients were randomized to the intervention and control groups at each center (trial protocol, 2005). Overall, 881 were randomized to the early ECV group and 883 to the delayed ECV group. The recruitment rates and balance of stratification are presented in Figure 2.

**Figure 3:**
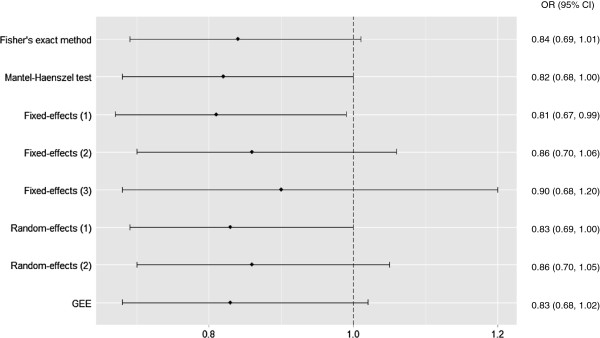
**Forest plot for Outcome 1: cesarean section.** Mantel-Haenszel test included 78 centers and 1761 participants. Fixed-effects (1): logistic regression with fixed center effect and fixed treatment effect, included 71 centers and 1739 participants. Logistic regression with fixed center effect and fixed treatment-by-center interaction, both weighted (Fixed-effects (2)) and un-weighted by center size (Fixed-effects (3)), included 57 centers and 1655 participants. Random-effects (1): logistic regression with random center effect and fixed treatment effect, Random-effects (2): logistic regression with random center effect and random treatment-by-center interaction, and GEE used all 81 centers and 1764 participants. CI, confidence interval, GEE, generalized estimating equations, OR, odds ratio.

**Figure 4:**
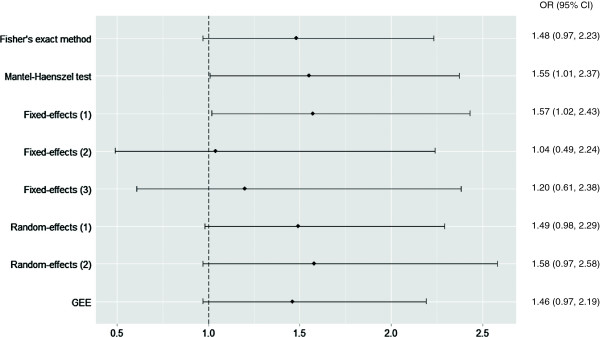
**Forest plot for Outcome 2: preterm birth.** Mantel-Haenszel test included 78 centers and 1761 participants. Fixed-effects (1): logistic regression with fixed center effect and fixed treatment effect, included 46 centers and 1434 participants. Logistic regression with fixed center effect and fixed treatment-by-center interaction, both weighted (Fixed-effects (2)) and un-weighted by center size (Fixed-effects (3)), included 14 centers and 646 participants. Random-effects (1): logistic regression with random center effect and fixed treatment effect, Random-effects (2): logistic regression with random center effect and random treatment-by-center interaction, and GEE used all 81 centers and 1764 participants. CI, confidence interval, GEE, generalized estimating equations, OR, odds ratio.

**Figure 5:**
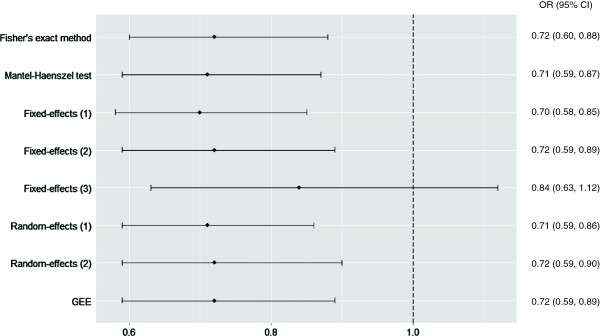
**Forest plot for Outcome 3: non-cephalic presentation at birth.** Mantel-Haenszel test included 78 centers and 1761 participants. Fixed-effects (1): logistic regression with fixed center effect and fixed treatment effect, included 71 centers and 1739 participants. Logistic regression with fixed center effect and fixed treatment-by-center interaction, both weighted (Fixed-effects (2)) and un-weighted by center size (Fixed-effects (3)), included 57 centers and 1649 participants. Random-effects (1): logistic regression with random center effect and fixed treatment effect, Random-effects (2): logistic regression with random center effect and random treatment-by-center interaction, and GEE used all 81 centers and 1764 participants. CI, confidence interval, GEE, generalized estimating equations, OR, odds ratio.

The seven statistical models described in the methods were applied to the EECV trial data to adjust for center effects, and the results for three selected outcomes are reported in Figures 3, 4, and 5.

### Low-recruiting centers

Different methodological approaches required different ways of handling low-recruiting, or ‘small’ centers in the analyses. The Mantel-Haenszel test used data from 78 out of 81 centers after the three centers that enrolled only one woman were removed from the analysis. For the three fixed-effects regressions, centers had to be removed from analysis if all the participants at that center were in one treatment group, or if all the participants experienced the same outcome. The removal of these centers was necessary because the statistical model is constructed only with centers that provide sufficient statistics [20]. Differing numbers of centers were removed from the analysis for the three outcomes of interest. A total of 71 centers representing 1739 participants were included for the outcomes of cesarean section and non-cephalic presentation at birth; 46 centers representing 1434 women were included for the outcome of preterm birth. Further centers were removed from analysis when the treatment-by-center interaction term was added to the fixed-effects regression model due to zero counts in the interaction term. For the outcome of cesarean section, 57 centers representing 1655 women were included, for preterm birth 14 centers representing 646 women were included, and for non-cephalic presentation at birth, 57 centers representing 1649 women were included in the analysis. The models, including a random-effects term and the GEE, were run with the entire dataset. Sparse center data does not cause problems for the random-effects approach because parameter space does not increase with the number of centers [20].

### Performance of various approaches to adjust for center effect

#### Outcome 1: cesarean section

ECV has been shown to reduce the risk of cesarean section at term [25], but the EECV2 trial did not demonstrate a statistically significant difference in cesarean section rates when comparing early ECV with the routine practice of conducting ECV at term [14]. The merged dataset was analyzed using Fisher’s exact test and associated exact CI estimation to provide an individual-level baseline analysis to which the methods that account for center could be compared. Unadjusted for center, the OR for cesarean section for those in the early ECV group was 0.84 (95% CI 0.69 to 1.01; *P* = 0.07).

Using methods to adjust for center effect gave estimates of effect that were similar to the unadjusted results (Figure 3). The seven models that adjusted for center effect provided ORs varying from 0.81 to 0.90, with lower limit 95% CI varying from 0.67 to 0.70 and upper limit 95% CI varying from 0.99 to 1.20. The least efficient model was the logistic regression with fixed treatment-by-center interaction term, un-weighted by center size, as shown by the wider 95% CI of 0.68 to 1.2.

#### Outcome 2: preterm birth

The OR of preterm birth for women in the early ECV group, unadjusted for center, was 1.48 (95% CI 0.97 to 2.23; *P* = 0.06).

There was some variation in the results obtained by using different models to account for center effect. In two models, the odds of preterm birth for women in the early ECV group reached statistical significance. These were the Mantel-Haenszel test (1.55; 95% CI 1.01 to 2.37) and the first fixed-effect model (logistic regression with fixed center effect and fixed treatment effect; 1.57; 95% CI 1.02 to 2.43). In contrast, in the models that included a fixed treatment-by-center interaction term (both weighted and un-weighted by center size), the point estimates are closer to the null value and the CIs are wider, but the results are based on data from only 14 centers. As shown in Figure 4, the overall results for the preterm birth outcome do not indicate that the adjusted results are very different from the unadjusted results.

#### Outcome 3: non-cephalic presentation at birth

Unadjusted for center, the OR of having a baby in a non-cephalic presentation at the time of birth was 0.72 (95% CI 0.60 to 0.88; *P* = 0.001) for women in the early ECV group compared to the delayed ECV group.

The adjusted results confirm that those in the early ECV group were more likely to have a cephalic presentation at the time of birth and indicate robust results under different methods of adjusting for center (Figure 5). The OR varied from 0.70 to 0.72 among six out of seven statistical models. The seventh model, logistic regression with fixed center effect and fixed treatment-by-center interaction un-weighted by center size, resulted in much wider CIs and a non-significant result. This deviation is likely due to the equal weighting of small centers with larger centers.

#### Magnitude of between-center differences

ICC was calculated for each of the three outcomes to quantify the average correlation between outcomes within the same center. For cesarean section, the ICC was 0.036, for preterm delivery the ICC was 0.014, and for non-cephalic presentation at birth the ICC was 0.048. These values suggest that the power to detect a treatment effect could be underestimated by approximately 0.5 to 4.0% [23].

## Discussion

Seven statistical models, available in the statistics literature, were applied to data from the EECV trials to provide researchers with an empirical example of accounting for center in a multicenter trial with binary outcomes. While population inference is the goal of clinical research, assuming that individuals recruited to a center are independent of others at the same centre may reduce the statistical power needed to show a treatment effect when the clustering of outcomes occurs. Our results show how changing statistical assumptions can confirm the magnitude of the treatment effect and builds on previous works addressing statistical analysis in multicenter trials [8, 26, 27].

As we applied statistical models to adjust for center effect, issues arose that may be of interest to researchers attempting similar adjustment. Low-recruiting centers were problematic in the application of fixed-effects logistic regression models. Centers that contain only patients of one treatment group or experiencing only one type of outcome event need to be removed. Since patients at small centers provide little information to overall treatment effect, removing them may not cause a problem [20] and this was confirmed in our results. However, when an interaction term is added to the equation, further centers need to be dropped, particularly for rare outcomes, and the resultant findings display low precision. We also illustrated the effect that weighting (or not weighting) by center size has on the logistic regression with fixed center effect and fixed treatment-by-center interaction. Our results for the un-weighted model have the widest CIs and most different effect estimates than any other model, confirming the work of Senn that weighting small and large centers equally can actually increase the variation around the estimate [17].

GEE was the one unconditional method applied to the dataset. GEE uses covariance matrices and/or structures to estimate correlation within centers that are inherently weighted to center size. GEE was well suited to the EECV trial data where there were many centers and the center size varied widely. The small difference between results from the GEE approach and the conditional methods may be partly explained by the non-collapsibility of ORs with non-null treatment effects [28].

Missingness in the EECV trial dataset used in this study was unlikely to have influenced the results of these analyses because it was not substantial. When the outcome is missing completely at random, GEE yields consistent estimators of the regression parameters, provided the model for the mean is correctly specified. Likelihood-based methods such as random-effects models may be a better alternative when missingness is at random [29–32]. When missingness depends on the unobserved outcomes, none of the methods can guarantee an unbiased estimate of effect.

This secondary analysis adds to the knowledge base regarding center effect in multicenter RCTs. A study by Kahan and Morris [33] looked at clustering in individual randomized trials by using both a case study and a simulation. They concluded that clustering by recruitment center is ‘non-ignorable’ when both patient outcomes and treatment assignments are correlated within clusters. Their simulation indicated that adjusting for center gave correct type 1 error rates. In future research trials, protocols ought to include plans to account for center effect, especially as starting with the sample size calculation as the magnitude of center effect can even change the sample size required [34].

## Conclusions

Awareness of the center effect in multicenter RCTs is growing as literature describing its occurrence accumulates. We conducted a secondary analysis of the EECV trials - large obstetric trials that included many centers with a wide range of recruitment rates in various international locales - to adjust for center effect. Our results did not change the overall conclusions of the EECV trials, however, adjusting for center effect increases confidence in the results by illustrating the robustness of the study findings under different statistical assumptions. This secondary analysis provides an example to support clinical researchers in their pursuit to adjust for center effect in the design, analysis, and interpretation of multicenter RCTs.

## Abbreviations

CONSORT: Consolidated standards of reporting trials
CI: Confidence interval
ECV: External cephalic version
EECV: Early External Cephalic Version
ICC: Intra-class correlation
GEE: Generalized estimating equation
OR: Odds ratio
RCT: Randomized controlled trial
RR: Relative risk.
